# POLQ inhibition attenuates the stemness and ferroptosis resistance in gastric cancer cells via downregulation of dihydroorotate dehydrogenase

**DOI:** 10.1038/s41419-024-06618-5

**Published:** 2024-04-04

**Authors:** Yanmei Peng, Wenbo Zheng, Yuehong Chen, Xuetao Lei, Zhijing Yang, Yuxuan Yang, Weiqi Liang, Kai Sun, Guoxin Li, Jiang Yu

**Affiliations:** 1 Department of General Surgery & Guangdong Provincial Key Laboratory of Precision Medicine for Gastrointestinal Tumor, Nanfang Hospital, The First School of Clinical Medicine, Southern Medical University, Guangzhou, Guangdong 510515 China; 2 Beijing Tsinghua Changgung Hospital, School of Clinical Medicine, Tsinghua University, Beijing, 102218 China

**Keywords:** Cancer stem cells, Gastric cancer

## Abstract

Gastric cancer (GC) contains subpopulations of cancer stem cells (CSCs), which are described as the main contributors in tumor initiation and metastasis. It is necessary to clarify the molecular mechanism underlying CSCs phenotype and develop novel biomarkers and therapeutic targets for gastric cancer. Here, we show that POLQ positively regulates stem cell-like characteristics of gastric cancer cells, knockdown of POLQ suppressed the stemness of GC cells in vitro and in vivo. Further mechanistic studies revealed that POLQ knockdown could downregulate the expression of dihydroorotate dehydrogenase (DHODH). DHODH overexpression rescued the reduced stemness resulted by POLQ knockdown. Furthermore, we found that POLQ expression correlated with resistance to ferroptosis, and POLQ inhibition renders gastric cancer cells more vulnerable to ferroptosis. Further investigation revealed that POLQ regulated DHODH expression via the transcription factors E2F4, thereby regulating ferroptosis resistance and stemness of gastric cancer cells. Given the importance of POLQ in stemness and ferroptosis resistance of GC, we further evaluated the therapeutic potential of POLQ inhibitor novobiocin, the results show that novobiocin attenuates the stemness of GC cells and increased ferroptosis sensitivity. Moreover, the combination of POLQ inhibitor and ferroptosis inducer synergistically suppressed MGC-803 xenograft tumor growth and diminished metastasis. Our results identify a POLQ-mediated stemness and ferroptosis defense mechanism and provide a new therapeutic strategy for gastric cancer.

## Introduction

Gastric cancer (GC) is the fifth most diagnosed malignancy and the fourth deadliest cancer worldwide [1]. Treatment strategies include surgery, chemo- or immunotherapy [2, 3]. However, a high percentage of GC patients eventually relapse, resulting in an overall 5-year survival rate of only 20–30%. Cancer stem cells (CSCs), a small population of cancer cells with stem cell-like properties, have been regarded as the root of tumor relapse, metastasis, and drug resistance [4–6]. Thus, it is urgent and necessary to clarify the molecular mechanism underlying CSCs phenotype and develop novel biomarkers and therapeutic targets for gastric cancer.

Polymerase theta (POLQ) is a widely conserved DNA polymerase that mediates a microhomology-mediated, error-prone, double-strand break (DSB) repair pathway, referred to as polymerase theta-mediated end joining (TMEJ) [7, 8]. In normal cells, TMEJ accounts for a small minority of DSB repair. POLQ inhibitors, such as novobiocin, have been developed for anticancer therapy [9, 10]. However, the role of POLQ in stemness of GC cells is unclear.

Ferroptosis, a form of regulated cell death induced by excessive lipid peroxidation, has recently emerged as a key tumor suppression mechanism [11, 12]. Glutathione peroxidase 4 (GPX4), ferroptosis suppressor protein 1 (FSP1), and dihydroorotate dehydrogenase (DHODH) constitute three major ferroptosis defense systems [13–15]. Although cancer cells usually have a higher level of ROS, which is essential for tumor progression, such as migration, proliferation, and survival, while CSCs are believed to possess a lower level of ROS [16]. Recent studies reported that cancer stem-like cells can escape ferroptosis by upregulated an antioxidant response [17].

Herein, we found that POLQ positively regulates stem cell-like characteristics and ferroptosis resistance of gastric cancer cells. POLQ inhibition suppressed the stemness of GC cells and increase ferroptosis sensitivity in vitro and in vivo. Further mechanistic studies revealed that POLQ inhibition could downregulate the expression of DHODH. Moreover, the combination of POLQ inhibitor and ferroptosis inducer synergistically suppressed MGC-803 xenograft tumor growth and diminished metastasis.

## Methods

### Cell lines and culture

Human GC cell lines (AGS and MGC-803) were obtained from Chinese Academy of Sciences (Shanghai, China). These cell lines and their derived cell lines were cultured in DMEM (Gibco) with 10% Fetal bovine serum (Procell Life Science &Technology) at 37 °C and 5% CO_2_.

### Generation of shRNA expression cells

Lentiviral vectors plasmids were constructed by GeneCopoeia, Inc (China). shPOLQ cells were generated by infecting AGS and MGC-803 cells with indicated shPOLQ lentiviruses and selecting with 5 μg/ml puromycin. shNC cells were generated by infecting indicated cells with shNC lentiviruses. ShPOLQ target sequences was CCTTAAGACTGTAGGTACTAT.

### Generation of stable expression cells

To generate DHODH overexpression cells, indicated cells were infected with PLVX-IRES-DHODH lentiviruses. Cells were selected with 1000 μg/ml hygromycin B.

### Animal models

Animal experiments were performed according to the approved procedures by the Southern Medical University Institutional Animal Care and Use Committee. The 5-week-old male BALB/c nude mice were purchased from the Central Laboratory of Animal Science at Southern Medical University (Guangzhou, China). In vivo limiting dilution assay was performed using MGC-803 cells. POLQ-knockdown cells and control cells were diluted into different concentrations (5 × 10^6^, 1 × 10^6^ and 2 × 10^5^ cells per 100 µl) and injected subcutaneously (*n* = 5 per group) for two weeks. In vivo bioluminescent imaging was performed to determine the tumor incidence. Mice were injected i.p. with 100 μl of d-luciferin (15 mg/ml in PBS) and imaged 10 min after injection using a BLT AniView Phoenix system. The frequency of stem cells was assessed by Extreme Limiting Dilution Analysis Program.

For Therapeutic experiments, athymic nude mice were injected subcutaneously with 1 × 10^7^ MGC-803 cells (n = 4 per group). When the tumors reached 50–100 mm^3^ in volume, the mice were assigned randomly into different treatment groups for the treatment of vehicle, novobiocin, sulfasalazine, or sulfasalazine in combination with novobiocin. Novobiocin and sulfasalazine were prepared fresh in PBS each time before IP injection and intraperitoneally injected daily at a dose of 100 mg/kg. Thirty days post-cell injection, mice were euthanized and tumors were dissected for weight, qRT-PCR, histological and immunostaining analysis. For the intraperitoneal tumor formation model, five million MGC-803 cells were injected into the lower right abdominal cavity of each mouse (*n* = 4 per group). The mice were assigned randomly into different treatment groups for the treatment of vehicle, novobiocin, sulfasalazine, or sulfasalazine in combination with novobiocin. Novobiocin and sulfasalazine intraperitoneally injected daily at a dose of 100 mg/kg. After two weeks of treatment, in vivo bioluminescent imaging was performed to determine the tumor incidence.

### Cell viability assay

Cells were seeded in 96 well plates (5000 cells per well) and incubated with indicated treatments. Cell viability was determined by the Cell Counting Kit-8. The absorbance at 450 nm (OD450) of the cells was measured using a microplate reader.

### Mammosphere assay

GC cells were digested from the tissue culture plate and washed with PBS. Then, single-cell suspension cells (5000 cells per well) were plated on 6-well Ultra-Low Attachment Plates and cultured in DMEM/F12 (1:1) medium containing 1 × B27, 20 ng/ml bFGF and 20 ng/ml EGF for 10 days. Oncospheres were observed and captured by inverted microscopy.

### Immunoblotting

Cell pellets were lysed using RIPA lysis buffer (FUDE Biological Technology) and the protein concentration was determined by a BCA protein assay. 30 μg of protein was used for immunoblot analysis using antibodies against DHODH (1:1000; Proteintech), POLQ (1:1000; Affinity), 4HNE (1 µg/ml; Jaica), and β-actin (1:1000; Proteintech).

### Lipid peroxidation assay

Cells were incubated in a 6-well plates containing 5 μM BODIPY-581/591 C11 dye (Invitrogen, D3861). After incubation at 37 °C for 20 min, cells were washed twice with PBS. For imaging, cells were imaged at 40× magnification using a Zeiss LSM980. For flow cytometry analysis, cells were trypsinized, filtered into single-cell suspensions and then transferred to FACS tubes. Lipid peroxidation was assessed using the flow cytometer CytoFLEX with a 488 nm laser. A minimum of 10,000 single cells were analyzed per well.

### Quantitative real-time PCR (qRT-PCR)

Total RNA from cultured cells was extracted using the RNA-Quick Purification Kit (ESscience). Quantitative real-time PCR (qPCR) assays were carried out to detect mRNA expression using the HiScript® II QRT SuperMix for qPCR (R222-01; Vazyme) and ChamQ SYBR qPCR Master Mix (Q311-02; Vazyme) according to the manufacturer’s instructions. β-actin was used as an internal control. The primers are listed in Table S1.

### Immunohistochemical staining

Mouse xenograft tumors were collected at the end of treatment and immediately fixed in 10% neutral buffered formalin. Samples were subjected to embedding in paraffin and sectioning at 5 µm. The resulting sections were used for haematoxylin and eosin (H&E) and immunohistochemistry staining. The following primary antibodies were used: DHODH (1:100; Proteintech), 4HNE (5 µg/ml; Jaica), and Ki67(1:100; Proteintech). A goat anti-rabbit IgG and a goat anti-mouse IgG were used as secondary antibodies. Immunohistochemical staining was performed using a Vector DAB kit.

### Transcriptomic analysis

RNA from MGC-803-ShNC and MGC-803-ShPOLQ was extracted using TRIzol reagent. The purity of the obtained RNA was determined by NanoPhotometer ® (IMPLEN, CA, USA). The concentration and integrity of RNA samples were detected by Agilent 2100 RNA nano 6000 assay kit (Agilent Technologies, CA, USA). High-quality RNA was used for cDNA library construction and Illumina sequencing with commercial service by AccuraMed Technology Limited Co., Ltd (Guangzhou, China). One to three micrograms total RNA per sample was used to construct a transcriptome sequencing library. The VAHTS Universal V6 RNA-Seq Library Prepkit for Illumina was used with different index tags for database establishment. The process included mRNA enrichment, fragmentation, first-strand cDNA synthesis, RNaseH degradation, second-strand cDNA synthesis, and purification. Terminal repair, A-tail addition, sequencing linker connection, fragment size selection, and PCR amplification were performed to obtain the final cDNA library. Library concentration was measured using Qubit® RNA Assay Kit in Qubit® 3.0, insert size assessed with the Agilent Bioanalyzer 2100 system (Agilent Technologies, CA, USA), and effective concentration quantified with Bio-RAD CFX 96 fluorescence quantitative PCR. After qualification, samples were pooled based on effective concentration and sequencing was done on the Illumina platform using the PE150 strategy for 150-bp double-ended reads. Sequencing followed the Sequencing by Synthesis principle. Four fluorescently labeled dNTPs, DNA polymerase, and linker primers were added to the flow cell for amplification. As each sequencing cluster extended a complementary chain, fluorescently labeled dNTPs were added to release corresponding fluorescence. A sequencer captured sequence information by detecting fluorescence signals, and computer software converted the signals into sequencing peaks. The normalized expression levels of different samples were converted to FPKM (Fragments Per Kilobase of transcript per Million mapped fragments). RNA-seq data is listed in Table S2.

### Generation of luciferase expression cells

The luciferase viruses with a GFP tag were purchased from Genechem (Shanghai, China). MGC-803 GC cells were transduced with luciferase lentivirus for 48 h and then selected by fluorescence activated cell sorting.

### Statistics and reproducibility

All data were obtained at least three independent experiments or samples. Statistical analyses were performed using two tailed t-test for pairwise comparison and one-way or two-way analysis of variance (ANOVA) for multiple comparisons on GraphPad Prism 9. Data are presented as means ± standard deviation (SD). *n* values indicate biologically independent samples and experiments. *P < 0.05; **P < 0.01; ***P < 0.001; ****P < 0.0001; ns., non-significant.

## Results

### **POLQ positively regulates stem cell-like characteristics of gastric cancer cells**

**Fig. 1:**
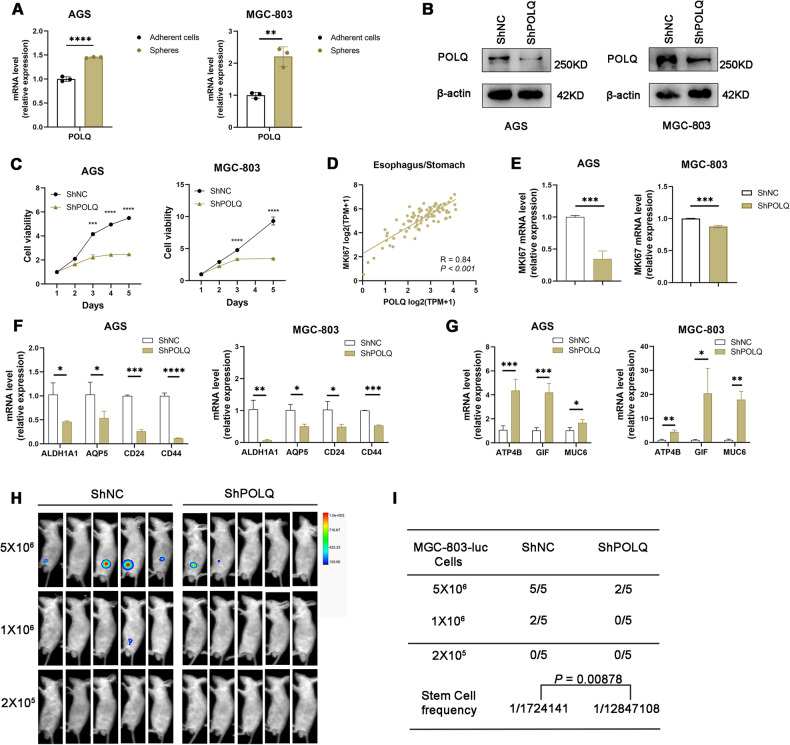
POLQ positively regulates stem cell-like characteristics of gastric cancer cells. **A** The levels of POLQ were detected in adherent cells and spheres derived from AGS and MGC-803 cells using qRT-PCR. **B** POLQ protein levels in AGS and MGC-803 cells expressing shRNA or shPOLQ. **C** Cell proliferation of control and POLQ- knockdown cell lines were determined using CCK-8 assay. **D** Public database [https://depmap.org/portal/] correlation analysis in cell lines (n = 84) between POLQ and MKI67. Significance was evaluated by Pearson correlation. **E** The mRNA levels of MKI67 were detected in control and POLQ- knockdown cell lines. The mRNA levels of gastric cancer stem cell markers (**F**) and differentiated cell markers (**G**) in control and POLQ- knockdown cell lines. **H** A series of limiting diluted POLQ-knockdown cells and their corresponding control cells were subcutaneously inoculated into nude mice. Bioluminescent imaging (BLI) was performed. **I** The CSC frequency was calculated using ELDA software. Results are shown as mean ± SD. *P < 0.05; **P < 0.01; ***P < 0.001.

Previous studies have indicated that sphere-forming cells derived from gastric cancer cell lines through serum-free culture methods exhibit stem cell-like characteristics [18, 19]. To clarify the role of POLQ in the stemness of GC cells, we first analyzed the expression of *POLQ* and stem cell markers *ALDH1A1*, *AQP5*, *CD24*, and *CD44* in spheres and adherent cells. Our results showed that *POLQ*, *ALDH1A1*, *AQP5*, and *CD44* were abundantly expressed in sphere cells from GC cell lines (Figs. 1A and S1A). Next, we created stable AGS and MGC-803 cell lines expressing either a nontargeting shRNA control or shRNA to POLQ and determined whether expression of POLQ had a direct role in regulating the stemness of GC cells. Successful POLQ knockdown was verified with immunoblot (Fig. 1B). Our results showed that knockdown of POLQ significantly suppressed the proliferation in these cells (Fig. 1C). Moreover, analysis of public database (DepMap) revealed co-expression of *MKI67* and *POLQ* in GC cell lines (Fig. 1D). POLQ knockdown decreased the mRNA expression levels of *MKI67* in GC cells (Fig. 1E). We also found that mRNA of stemness markers *ALDH1A1*, *AQP5*, *CD24* and *CD44* were reduced in POLQ knockdown GC cells (Fig. 1F). mRNA of differentiated cell markers *ATP4B*, *GIF*, and *MUC6* were increased (Fig. 1G). Next, we performed in vivo limiting dilution assays (LDAs) to monitor the effect of POLQ on the tumor-initiating capacity of GC cells. Three doses (5 × 10^6^, 1 × 10^6^ and 2 × 10^5^) of POLQ-knockdown cells and their corresponding control cells were subcutaneously inoculated into nude mice. We found that POLQ-knockdown cells displayed lower tumorigenicity than the control cells (Fig. 1H, I). Collectively, these data suggest that POLQ positively regulates the stem-like characteristics of GC cells.

### POLQ regulates the cancer stem cell-like characteristics of GC cells through DHODH

**Fig. 2:**
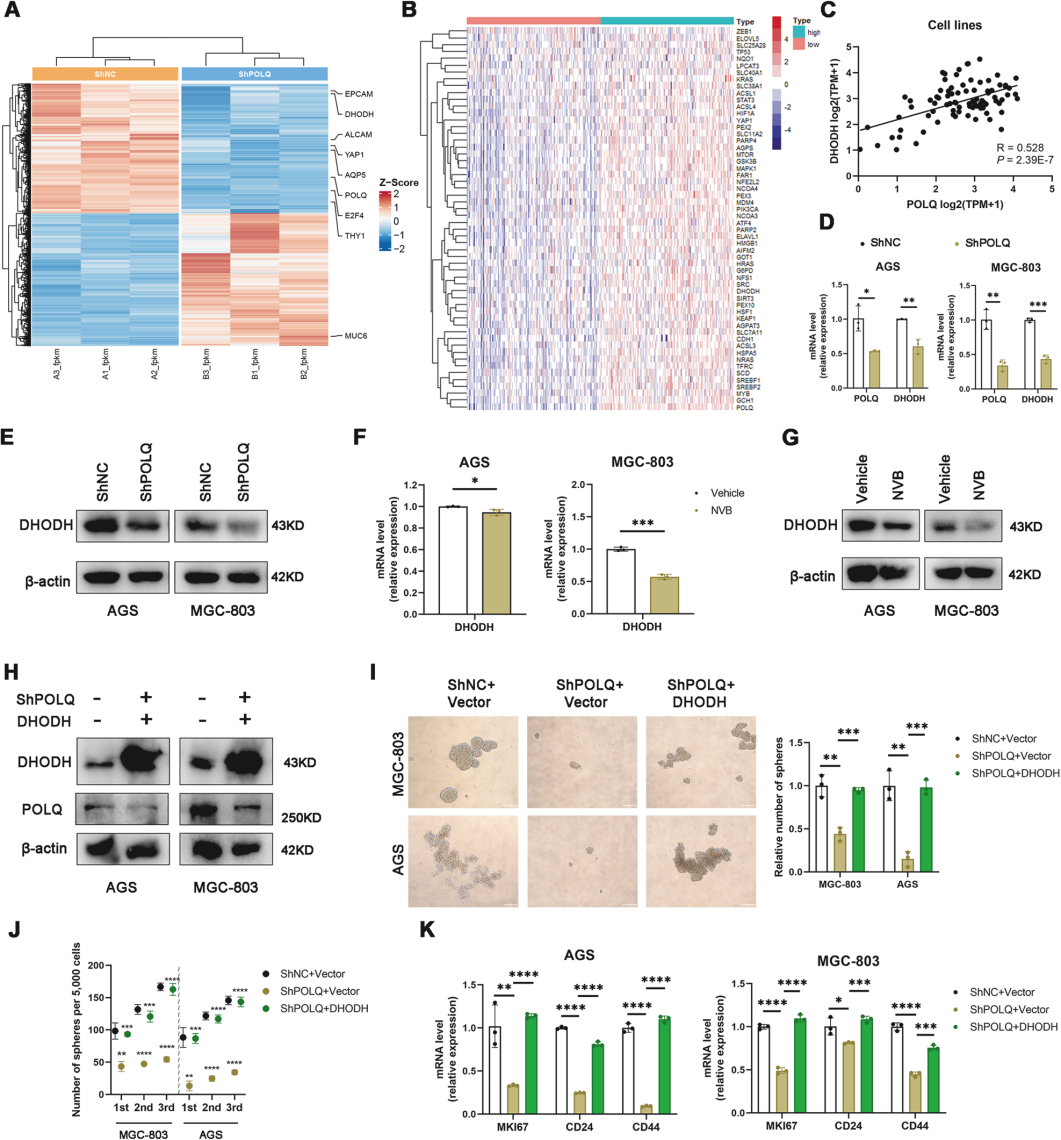
POLQ regulates the cancer stem cell-like characteristics of GC cells through DHODH. **A** Heatmap showing differently expressed genes in control and POLQ-knockdown GC cells. Values are scaled as indicated (2 to −2; *n* = 3). **B** Heatmap showing differently expressed genes in POLQ^low^ versus POLQ^high^ stomach adenocarcinoma. **C** Public database [https://depmap.org/portal/] correlation analysis in cell lines (*n* = 84) between POLQ and DHODH. Significance was evaluated by Pearson correlation. **D**, **E** The mRNA and protein levels of DHODH were detected in control and POLQ- knockdown cell lines. **F**, **G** The mRNA and protein levels of DHODH were detected in GC cells treated with vehicle or NVB (100 µM) for 24 h. **H** Protein expression levels in POLQ- knockdown cells overexpressing DHODH. **I** Sphere formation in control (ShNC), POLQ knockdown (ShPOLQ), and ShPOLQ + DHODH cells was analyzed using oncosphere-initiating medium. **J** The serial sphere-forming capacity. **K** The mRNA levels of gastric cancer stem cell markers in control (ShNC), POLQ knockdown (ShPOLQ), and ShPOLQ + DHODH cells. Results are shown as mean ± SD. *P < 0.05; **P < 0.01; ***P < 0.001.

To interrogate how POLQ regulates stemness of GC cells, we profiled the transcriptomes and identified the fold‐change of differentially expressed genes in POLQ-knockdown GC cells. Our results revealed that compared to normal control GC cells, *AQP5*, *THY1 (CD90)*, *ALCAM (CD166)*, *EPCAM*, *YAP1*, *DHODH*, and *E2F4* were significantly downregulated in POLQ-knockdown GC cells. These genes had been previously implicated in cancer cells stemness [20–24]. Additionally, the differentiation marker *MUC6* was upregulated in POLQ-knockdown GC cells (Fig. 2A). We performed the gene-set enrichment analysis (GSEA) of differential expressed genes (DEGs) between MGC-803-ShPOLQ and MGC-803-ShNC cells. Among the hallmark signature gene sets, control group was found to strongly associate with Hedgehog signaling pathway, Notch signaling pathway, and Wnt signaling pathway (Figure S1B). These pathways had been previously implicated in gastric cancer stemness [25]. We further analyzed genes mRNA expression with TCGA (The Cancer Genome Atlas) database and found that the expression level of *DHODH* was also higher in POLQ^high^ than POLQ^low^ human gastric cancer (*n* = 343) (Fig. 2B). Moreover, analysis of public database (DepMap) revealed that expression level of *POLQ* was positively correlated with *DHODH* in GC cell lines (Fig. 2C). We confirmed their expression level in POLQ-knockdown GC cells using quantitative reverse‐transcription PCR (qRT‐PCR) (Fig. 2D). Therefore, we hypothesized that POLQ regulates stemness in gastric cancer cells by modulating the expression of DHODH. Our results showed that POLQ depletion decreased DHODH expression by western blot (Fig. 2E). Consistent with observations of genetic inhibition of POLQ, POLQ inhibitor novobiocin (NVB) treatment decreased DHODH expression at both mRNA and protein levels (Fig. 2F, G). To clarify the role of DHODH in the stemness of GC cells, we detected the expression of *DHODH* in the spheres and adherent cells and the results showed that *DHODH* was abundantly expressed in sphere cells from GC cell lines (Fig. S1C). Next, to determine whether POLQ regulation of DHODH was crucial for POLQ‐induced tumor stemness, we performed spheroid formation assay using a stable GC cell line knockdown POLQ and overexpressing DHODH (Fig. 2H). We found that the reduced spheroid formation ability resulted by POLQ knockdown was rescued by DHODH overexpression (Fig. 2I, J). Furthermore, the stemness markers led by POLQ knockdown was reversed by DHODH overexpression (Fig. 2K). Hence, our results demonstrated that downregulation of DHODH was essential for POLQ inhibition to attenuates GC cell stemness.

### Targeting POLQ renders gastric cancer cells more vulnerable to ferroptosis

**Fig. 3:**
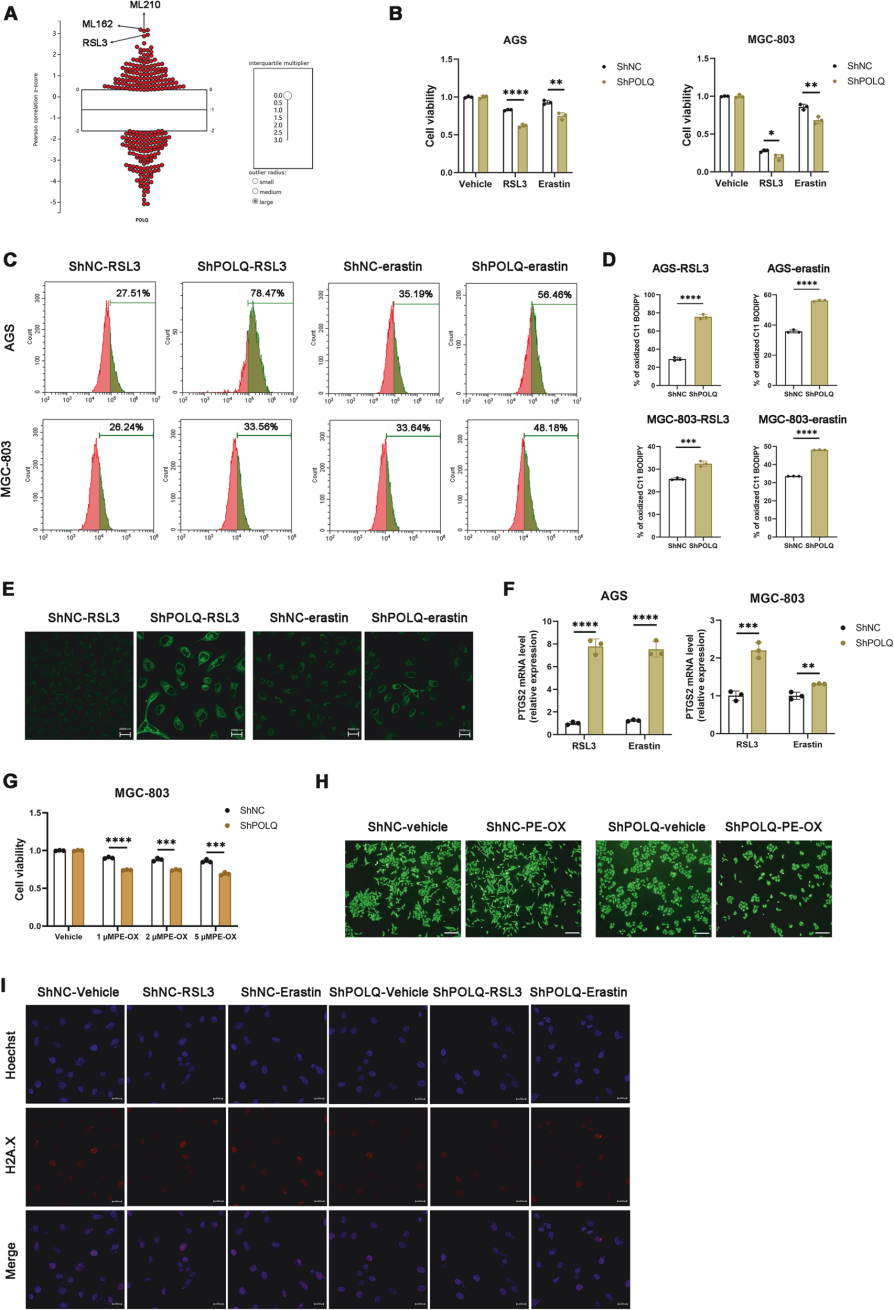
Targeting POLQ renders gastric cancer cells more vulnerable to ferroptosis. **A** High POLQ expression correlates with resistance to ferroptosis inducers (RSL3, ML162, and ML210) in cancer cells. Plotted data were mined from the CTRP database. **B** Cell viability in control and POLQ- knockdown GC cells treated with RSL3 (10 µM) or erastin (15 µM) for 24 h. **C**, **D** Respective lipid peroxidation levels were assessed and calculated by flow cytometry using BODIPY C11. **E** Control and POLQ- knockdown GC cells were collected for lipid peroxidation measurement (scale bar = 20 μm). **F** The mRNA levels of PTGS2 were detected in control and POLQ- knockdown GC cells treated with RSL3 (5 µM) or erastin (10 µM) for 24 h. **G** Cell viability in control and POLQ- knockdown GC cells treated with 1-stearoyl-2-15(S)-HpETE-sn-glycero-3-PE for 24 h. **H** Control and POLQ- knockdown GC cells were incubated with vehicle or 1-stearoyl-2-15(S)-HpETE-sn-glycero-3-PE for 24 h and stained with Calcein AM reagent and the representative images were acquired by fluorescence microscope (scale bar = 200 μm). **I** Cells were fixed and stained for γ–H2AX, 24 h after treated with vehicle, RSL3 or erastin (scale bar = 20 μm). Results are shown as mean ± SD. *P < 0.05; **P < 0.01; ***P < 0.001.

Since DHODH is a critical suppressor of ferroptosis through reducing ubiquinone (CoQ) to ubiquinol (CoQH_2_) [14, 26, 27], we wondered whether POLQ is involved in suppressing ferroptosis, we systematically interrogated the Cancer Therapeutics Response Portal datasets, we found that POLQ expression correlated with resistance to ferroptosis inducers ((RSL3, ML210 and ML162, which inhibit GPX4 activity) (Fig. 3A). We performed the gene-set enrichment analysis (GSEA) of differential expressed genes (DEGs) between MGC-803-ShPOLQ and MGC-803-ShNC cells and found that MGC-803-ShPOLQ group positively correlated with Ferroptosis pathway (Fig. S1D). In line with this, reduction in POLQ significantly promoted RSL3 and erastin-induced cell death (Fig. 3B). Furthermore, knockdown of POLQ notably elevated RSL3 and erastin-triggered lipid peroxidation and the levels of lipid metabolites, such as *PTGS2* levels (Fig. 3C–F). We also found that POLQ-knockdown GC cells were more sensitive to oxidized lipids 1-stearoyl-2-15(S)-HpETE-sn-glycero-3-PE than the control cells (Fig. 3G, H). These results indicate that POLQ inhibition renders gastric cancer cells more vulnerable to ferroptosis. Accumulated evidence demonstrates that POLQ is involved in the polymerase theta-mediated end joining (TMEJ), a DNA Double-Strand Break Repair Pathway [7, 8]. To investigate whether POLQ knockdown enhances DNA double-strand breaks to increase ferroptosis sensitivity of GC cells, we examined γH2AX foci formation on POLQ knockdown and control cells, both treated with vehicle or ferroptosis inducers, no significant difference in γH2AX foci formation was observed between these cells (Fig. 3I). This data suggests that the enhanced sensitivity of ferroptosis in POLQ-knockdown cells is not due to faulty DNA repair mechanisms, a possible explanation is that the homologous recombination function of these gastric cancer cells is normal.

### Upregulation the expression of DHODH blocks ferroptosis susceptibility mediated by POLQ inhibition

**Fig. 4:**
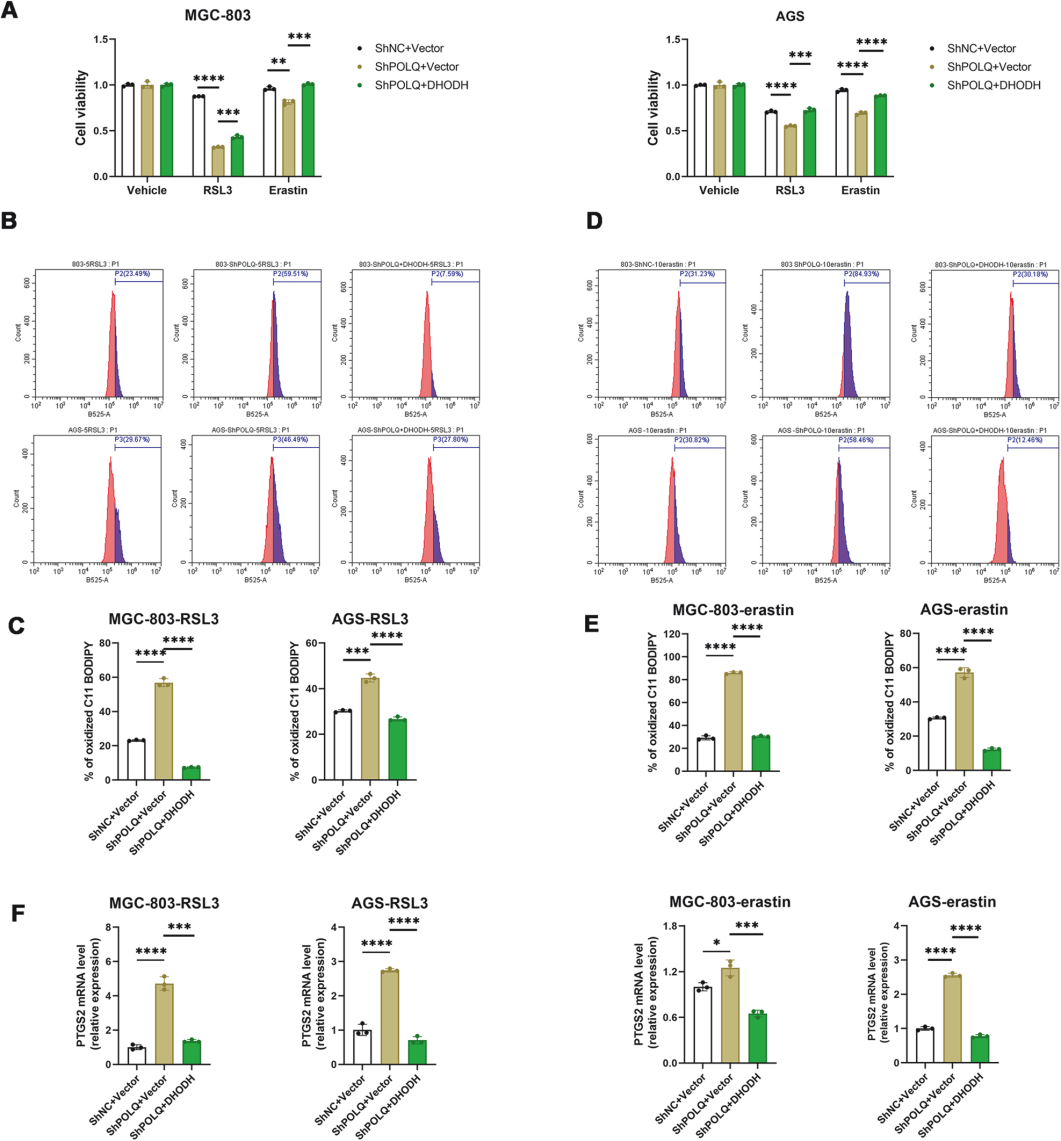
Upregulation the expression of DHODH blocks ferroptosis susceptibility mediated by POLQ inhibition. **A** Cell viability in control (ShNC), POLQ knockdown (ShPOLQ), and ShPOLQ + DHODH cells treated with RSL3 (10 µM) or erastin (15 µM) for 24 h. **B**-**E** Respective lipid peroxidation levels were assessed and calculated by flow cytometry using BODIPY C11. **F** The mRNA levels of PTGS2 were detected in control (ShNC), POLQ knockdown (ShPOLQ), and ShPOLQ + DHODH GC cells treated with RSL3 (5 µM) or erastin (10 µM) for 24 h. Results are shown as mean ± SD. *P < 0.05; **P < 0.01; ***P < 0.001.

To examine whether POLQ regulation of DHODH was crucial for POLQ‐induced tumor ferroptosis, examination of typical ferroptosis characteristics revealed that DHODH overexpression significantly prevented cell death caused by POLQ knockdown (Fig. 4A). At the same time, DHODH overexpression prevented the increase in RSL3 and erastin-triggered lipid peroxidation induced by POLQ knockdown (Fig. 4B–E). Moreover, the levels of lipid metabolites *PTGS2* levels induced by POLQ knockdown were prevented by DHODH overexpression (Fig. 4F). Hence, our results demonstrated that downregulation of DHODH was essential for POLQ inhibition to induce ferroptosis sensitivity.

### POLQ stimulates DHODH expression via the transcription factors E2F4

**Fig. 5:**
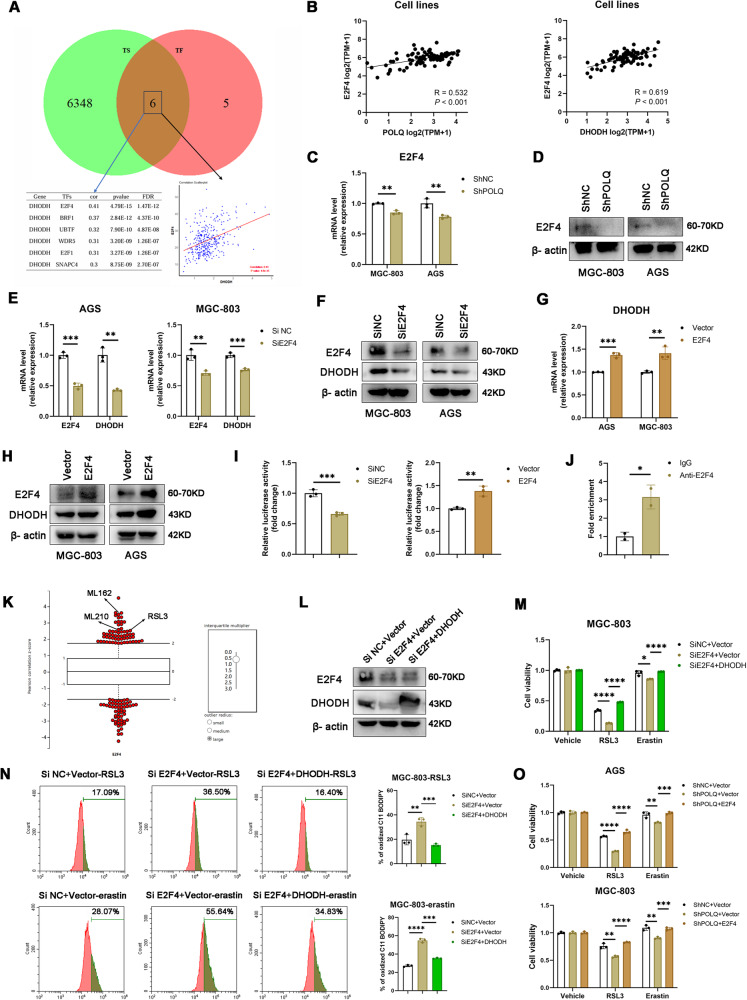
POLQ stimulates DHODH expression via the transcription factors E2F4. **A** Venn diagram showing a mode for screening the putative upstream TFs of DHODH. **B** Public database [https://depmap.org/portal/] correlation analysis in cell lines (n = 84) between E2F4, POLQ, and DHODH. Significance was evaluated by Pearson correlation. **C**, **D** The mRNA and protein levels of E2F4 were detected in control and POLQ- knockdown cell lines. **E**, **F** The mRNA and protein levels of E2F4 and DHODH were detected in control and E2F4- knockdown cell lines. **G**, **H** The mRNA and protein levels of DHODH were detected in control and E2F4 overexpression cell lines. **I** DHODH transcription activity was examined by luciferase assay. **J** ChIP results of E2F4 binding on DHODH promoter in MGC-803 cells, primers were designed based on E2F4 binding peak regions depicted in the JASPAR database. **K** High E2F4 expression correlates with resistance to ferroptosis inducers (RSL3, ML162, and ML210) in cancer cells. Plotted data were mined from the CTRP database. **L** Protein expression levels in E2F4- knockdown cells overexpressing DHODH. **M** Cell viability in control (Si NC), E2F4 knockdown (Si E2F4), and Si E2F4 + DHODH cells treated with RSL3 (10 µM) or erastin (15 µM) for 24 h. **N** Respective lipid peroxidation levels were assessed and calculated by flow cytometry using BODIPY C11. **O** Cell viability in control (ShNC), POLQ knockdown (ShPOLQ), and ShPOLQ + E2F4 cells treated with RSL3 (10 µM) or erastin (15 µM) for 24 h.

Next, we turned to explore the mechanism of POLQ regulating DHODH expression in GC cells. DHODH mRNA level decreased obviously in POLQ-knockdown cells, elucidating that DHODH was likely regulated by POLQ at transcriptional level. We analyzed the correlation between DHODH and all transcription factors, and intersected it with downregulated genes in the RNA-seq data from POLQ- knockdown and control MGC-803 cells. We found that E2F4 is the transcription factor with the strongest positive correlation with DHODH among the downregulated genes in the RNA-seq data (Fig. 5A). Moreover, analysis of public database (DepMap) revealed that expression level of *E2F4* was positively correlated with *POLQ and DHODH* in GC cell lines (Fig. 5B). Then, we confirmed E2F4 expression level in POLQ-knockdown GC cells using quantitative reverse‐transcription PCR (qRT‐PCR) and western blots. POLQ knockdown decreased E2F4 expression (Fig. 5C, D). We suppressed E2F4 expression by siRNAs in AGS and MGC-803 cells. Western blots and qPCR analysis indicated that, the knockdown of E2F4 inhibited the protein and mRNA expression of DHODH in GC cell lines (Fig. 5E, F). Besides, E2F4 overexpression was sufficient to activate endogenous DHODH expression in both mRNA and protein levels (Fig. 5G, H). We constructed a pGL3-DHODH promoter reporter gene system. Our data showed that the knockdown of E2F4 decreased the promoter-driven expression of luciferase. On the contrary, E2F4 overexpression enhanced the promoter-driven expression of luciferase (Fig. 5I). Furthermore, chromatin immunoprecipitation (ChIP) assay revealed that endogenous E2F4 readily bound to the DHODH promoter (Fig. 5J).

Next, we examined whether the knockdown of E2F4 induces a similar functional phenotype as POLQ-knockdown cells with respect to ferroptosis susceptibility and stemness. We systematically interrogated the Cancer Therapeutics Response Portal datasets, and found that E2F4 expression correlated with resistance to ferroptosis inducers ((RSL3, ML210 and ML162, which inhibit GPX4 activity) (Fig. 5K). Like the POLQ-knockdown cells, E2F4 knockdown resulted in increased ferroptosis sensitivity and lipid peroxidation in MGC-803 cells, which was significantly rescued by DHODH overexpression (Fig. 5L–N). In addition, knockdown of E2F4 decreased the stemness of GC cells, this effect was significantly rescued by DHODH overexpression (Fig. S1E). Furthermore, E2F4 overexpression restores resistance to ferroptosis and stemness in POLQ-knockdown AGS and MGC-803 cells (Figs. 5O and S1F, G). These findings establish that POLQ regulates DHODH expression via the transcription factor E2F4, thereby regulating ferroptosis resistance and stemness of gastric cancer cells.

### **Pharmacological inhibition of POLQ attenuates the stemness and ferroptosis resistance in gastric cancer cells**

**Fig. 6:**
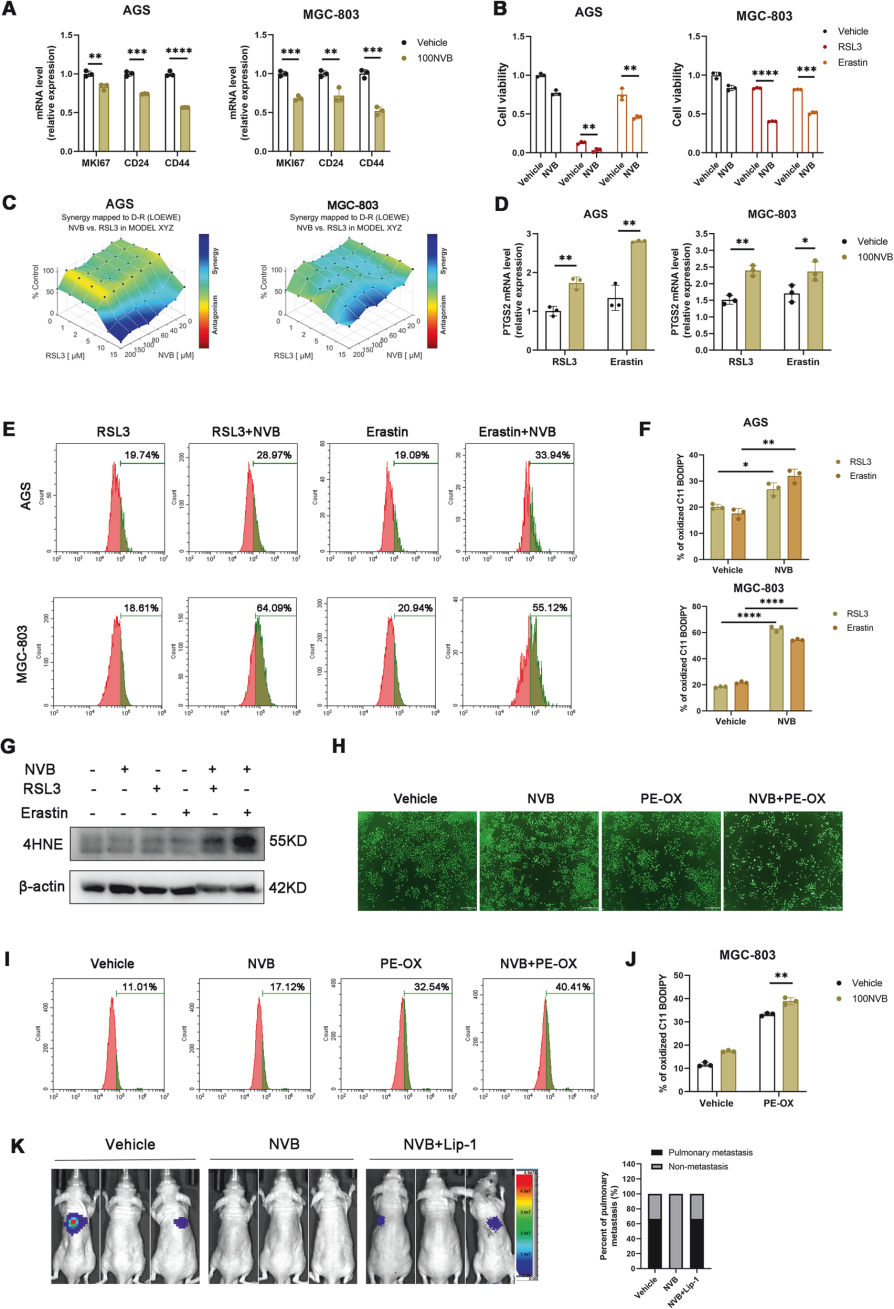
Pharmacological inhibition of POLQ attenuates the stemness and ferroptosis resistance in gastric cancer cells. **A** The mRNA levels of gastric cancer stem cell markers in GC cells treated with vehicle or NVB (100 µM) for 24 h. **B** Cell viability in GC cells treated with vehicle, RSL3, erastin or combined NVB for 24 h. **C** Analysis of combination of RSL3 and NVB in GC cells using combenefit software. **D** The mRNA levels of PTGS2 were detected in GC cells treated with RSL3, erastin or combined NVB for 24 h. **E**, **F** Respective lipid peroxidation levels were assessed and calculated by flow cytometry using BODIPY C11. **G** The protein levels of 4HNE were detected in GC cells treated with RSL3, erastin or combined NVB for 24 h. **H** MGC-803 cells were incubated with vehicle, 1-stearoyl-2-15(S)-HpETE-sn-glycero-3-PE or combined NVB for 24 h and stained with Calcein AM reagent and the representative images were acquired by fluorescence microscope (scale bar = 500 μm). **I**, **J** Respective lipid peroxidation levels were assessed and calculated by flow cytometry using BODIPY C11. **K** Left: BLI of the metastatic burden of mice intravenously injected with MGC-803-luc cells. The mice were treated with vehicle, NVB or combined Liproxstatin-1. Right: The proportion of pulmonary metastases. Results are shown as mean ± SD. *P < 0.05; **P < 0.01; ***P < 0.001.

The therapeutic potential of pharmacological POLQ inhibition in stemness and ferroptosis sensitivity of GC cells was further evaluated. We focused on novobiocin (NVB), a POLQ inhibitor which has been tested in multiple clinical trials [10]. We found that NVB treatment decreased the mRNA expression levels of *MKI67*, *CD24*, and *CD44* in GC cells (Fig. 6A). As expected, NVB treatment sensitized gastric cancer to ferroptosis (Fig. 6B). We also assessed the synergy of a combination of NVB and RSL3 treatment in AGS and MGC-803 cell lines, using the LOEWE synergy model [28]. The combination of NVB plus RSL3 resulted in synergistic cytotoxicity in both cell lines (Fig. 6C). Addition of NVB induced higher peroxidation levels in GC cells treated with RSL3 or erastin (Fig. 6D–G). Addition of NVB render GC cells more sensitive to oxidized lipids 1-stearoyl-2-15(S)-HpETE-sn-glycero-3-PE (Fig. 6H). Moreover, we found that addition of NVB induced higher peroxidation levels in GC cells treated with 1-stearoyl-2-15(S)-HpETE-sn-glycero-3-PE (Fig. 6I, J). Accumulated evidence demonstrates that ferroptosis inhibits metastasis by limited the survival of cancer cells in the blood [29, 30]. To determine if POLQ inhibition could reduce metastatic burden by increased ferroptosis, the tail vein metastasis model was utilized. After tail vein injection, the mice were treated with saline, NVB or combined Liproxstatin-1 as indicated. After a 4-week treatment, NVB diminished the pulmonary metastases derived from MGC-803 cells, liproxstatin-1 potently rescued this effect (Fig. 6K). Thus, pharmacological inhibition of POLQ might be a potential strategy against metastatic disease by sensitized cancer cells to ferroptosis.

### Sulfasalazine with POLQ inhibition efficiently facilitates treatment in gastric cancer

**Fig. 7:**
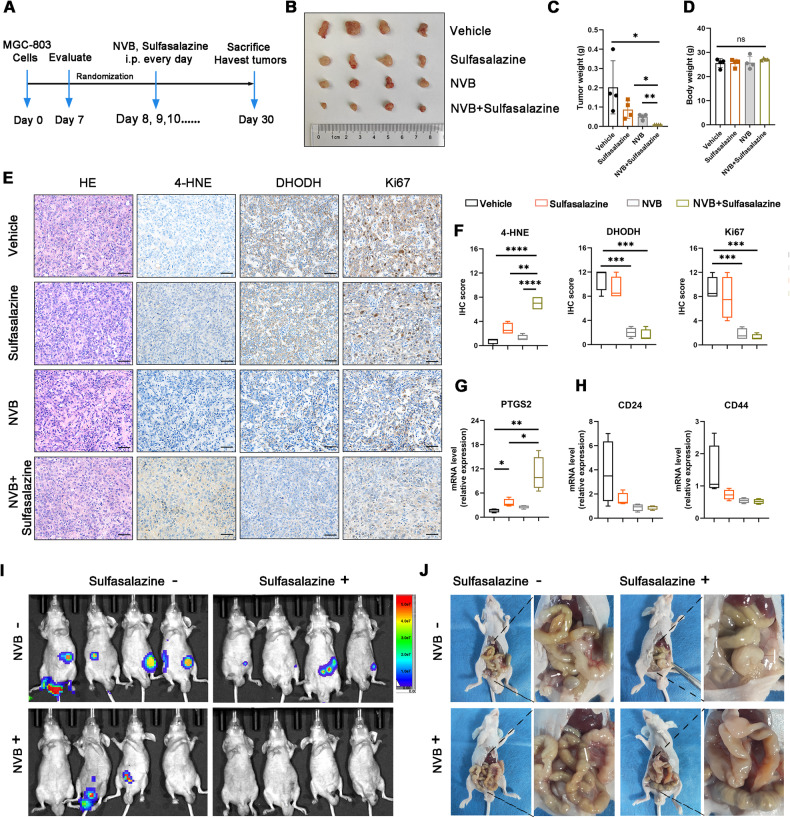
Sulfasalazine with POLQ inhibition efficiently facilitates treatment in gastric cancer. **A** Schematic of subcutaneous xenograft model treated with vehicle, novobiocin, sulfasalazine, or sulfasalazine in combination with novobiocin (*n* = 4). **B** Morphological images of the tumors. **C** The weights of subcutaneous tumors in indicated mice. **D** Mice weights were recorded after vehicle, novobiocin, sulfasalazine, or sulfasalazine in combination with novobiocin treatment. **E**, **F** IHC staining of DHODH, 4HNE, and MKi67 in subcutaneous tumors derived from indicated mice (scale bar = 50 μm). The expression of PTGS2 (**G**), CD24, and CD44 (**H**) in subcutaneous tumors from indicated mice. **I**, **J** BLI and images of intraperitoneal tumor formation model treated with vehicle, novobiocin, sulfasalazine, or sulfasalazine in combination with novobiocin (*n* = 4). Results are shown as mean ± SD. *P < 0.05; **P <0.01; ***P < 0.001; ns not significant.

Since pharmacological inhibition of POLQ attenuates the stemness of GC cells and sensitizes gastric cancer to ferroptosis, we further investigated the therapeutic potential of combined treatment with POLQ inhibitors and ferroptosis inducers in treating gastric tumors in vivo. We conducted animal experiments with a subcutaneous xenograft model and an intraperitoneal tumor formation model. For the subcutaneous xenograft model, combined treatment with POLQ inhibitors and sulfasalazine, a ferroptosis inducer that inhibits SLC7A11, synergistically suppressed MGC-803 xenograft tumor growth (Fig. 7A–C). Combined treatment with NVB and sulfasalazine dramatically inhibited the expression of Ki67 and DHODH, and increased the expression of 4HNE in the xenograft tissues, but not in control tumors and tumors treated with sulfasalazine alone (Fig. 7E, F). Furthermore, Combined treatment with NVB and sulfasalazine increased the mRNA expression of *PTGS2* (Fig. 7G). Despite the differences not being statistically significant, we can observe that the mRNA expression of stemness markers *CD24* and *CD44* were decreased in tumor tissues treated with NVB (*P* = 0.0687; *P* = 0.0796) (Fig. 7H). In all these animal studies, drug treatment did not significantly affect animal weights, suggesting that the treatment was well-tolerated in vivo (Fig. 7D).

For the intraperitoneal tumor formation model, five million MGC-803 cells were injected into the lower right abdominal cavity of each mouse. After two weeks of treatment, combined treatment with POLQ inhibitors and sulfasalazine decreased abdominal invasion (Fig. 7I, J). Together, our results suggest the combination of POLQ inhibitors with sulfasalazine as a potential agent to treat GC.

## Discussion

Increasing evidence suggests that cancer stem cells (CSCs) play a crucial role in the development of gastric cancer (GC) [25, 31]. CSCs possess the remarkable abilities of self-renewal and unlimited proliferation, which drive tumor formation. Additionally, CSCs also contribute significantly to the invasion and metastasis of gastric cancer [32]. While conventional tumor therapies effectively target a large number of tumor cells, they often fail to eradicate undifferentiated CSCs. The resistance of CSCs to existing therapies can be attributed to various mechanisms, including drug efflux by ABC transporters, activation of aldehyde dehydrogenase, overactivation of the DNA damage response (DDR) and apoptosis evasion [33, 34]. Their resistance to specific treatments may lead to the enrichment of CSCs during anti-tumor therapy, ultimately resulting in tumor recurrence [25]. Developing CSC-targeted therapies to prevent relapse or metastasis may be the key to successfully combating GC.

A crucial step in developing new therapeutic strategies targeting tumor-initiating cells in GC involves the molecular characterization of GCSCs. While the enzymatic activities of CD44 and ADLH (aldehyde dehydrogenase) are the most commonly used markers for identifying GCSCs, ongoing research is exploring other potential biomarkers, including CD24, CD54, CD326, LGR5, CD133, SOX2, OCT4, and NANOG [35–38]. However, the specificity of almost all explored markers remains uncertain [25]. Currently, the methods used to validate putative CSCs include xenotransplantation of tumors and sphere-forming assays in non-adherent cultures (containing epidermal growth factor and fibroblast growth factor 2 in serum-free medium) [4, 39]. In our study, we made a significant discovery regarding the role of POLQ in regulating the stemness of gastric cancer cells. POLQ, which is frequently overexpressed in many cancers and linked to a poorer prognosis, has garnered considerable attention from researchers due to its involvement in the double-strand break (DSB) repair pathway [8]. However, its precise role in GC progression remains largely unknown. In this novel study, we demonstrated, for the first time, that alterations in POLQ levels impact the phenotype and behavior of stemness characteristics in gastric cancer. Moreover, increasing evidence suggests that POLQ inhibitors, like novobiocin, can enhance chemotherapy sensitivity by suppressing homologous recombination and DNA double-strand break repair [10]. Intriguingly, our study also revealed an additional effect of POLQ inhibitors—reducing the stemness of gastric cancer cells.

Ferroptosis, a form of non-apoptotic cell death mediated by lipid peroxidation, has been shown to be resisted by tumor stem cells, which possess an enhanced antioxidant system [11, 17]. In our investigation, we uncovered that POLQ can confer resistance to ferroptosis by upregulating DHODH, one of the three major defense systems against ferroptosis that converts CoQ to CoQH_2_ [14]. By inhibiting POLQ, we effectively weakened the stemness and antioxidant capacity of gastric cancer cells, consequently heightening their sensitivity to ferroptosis. Sulfasalazine, a ferroptosis inducer widely used in treating conditions like rheumatoid arthritis, was examined in our study [40, 41]. Through subcutaneous tumor and peritoneal implantation models in nude mice, we found that POLQ inhibitors in combination with sulfasalazine synergistically exhibited anti-tumor effects, holding significant potential for direct translational applications in cancer therapy.

In conclusion, our results identify a POLQ-mediated stemness and ferroptosis defense mechanism by regulating E2F4-DHODH axis. This work suggests that the combination of POLQ inhibitor and ferroptosis inducer synergistically kill gastric CSCs and thus provides a new therapeutic strategy for gastric cancer.

### Supplementary information

Original Western Blots

Table S2

Supplementary material

## Data Availability

The supporting data of this study are available from the corresponding author upon reasonable request.
