# Supplementary figures and images for: POLQ inhibition attenuates the stemness and ferroptosis resistance in gastric cancer cells via downregulation of dihydroorotate dehydrogenase
Source: Cell Death Dis. 2024 Apr 4;15(4):248. doi: 10.1038/s41419-024-06618-5 (PMC10995193; doi:10.1038/s41419-024-06618-5)

**Figure 1**

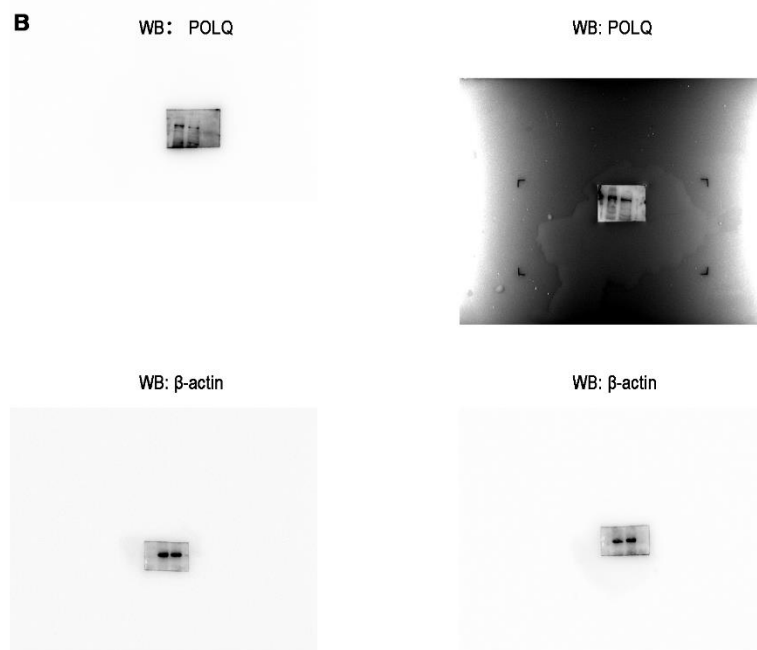

**Figure2**

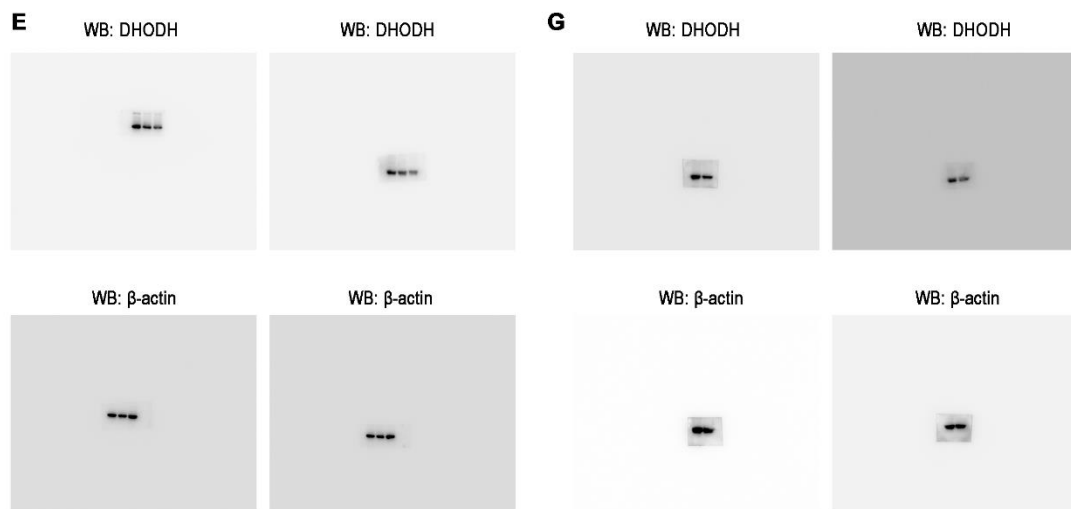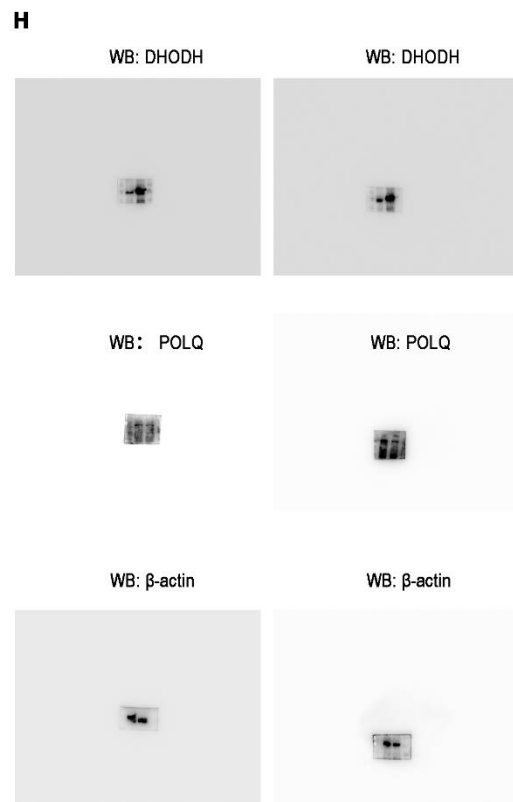

Figure 5

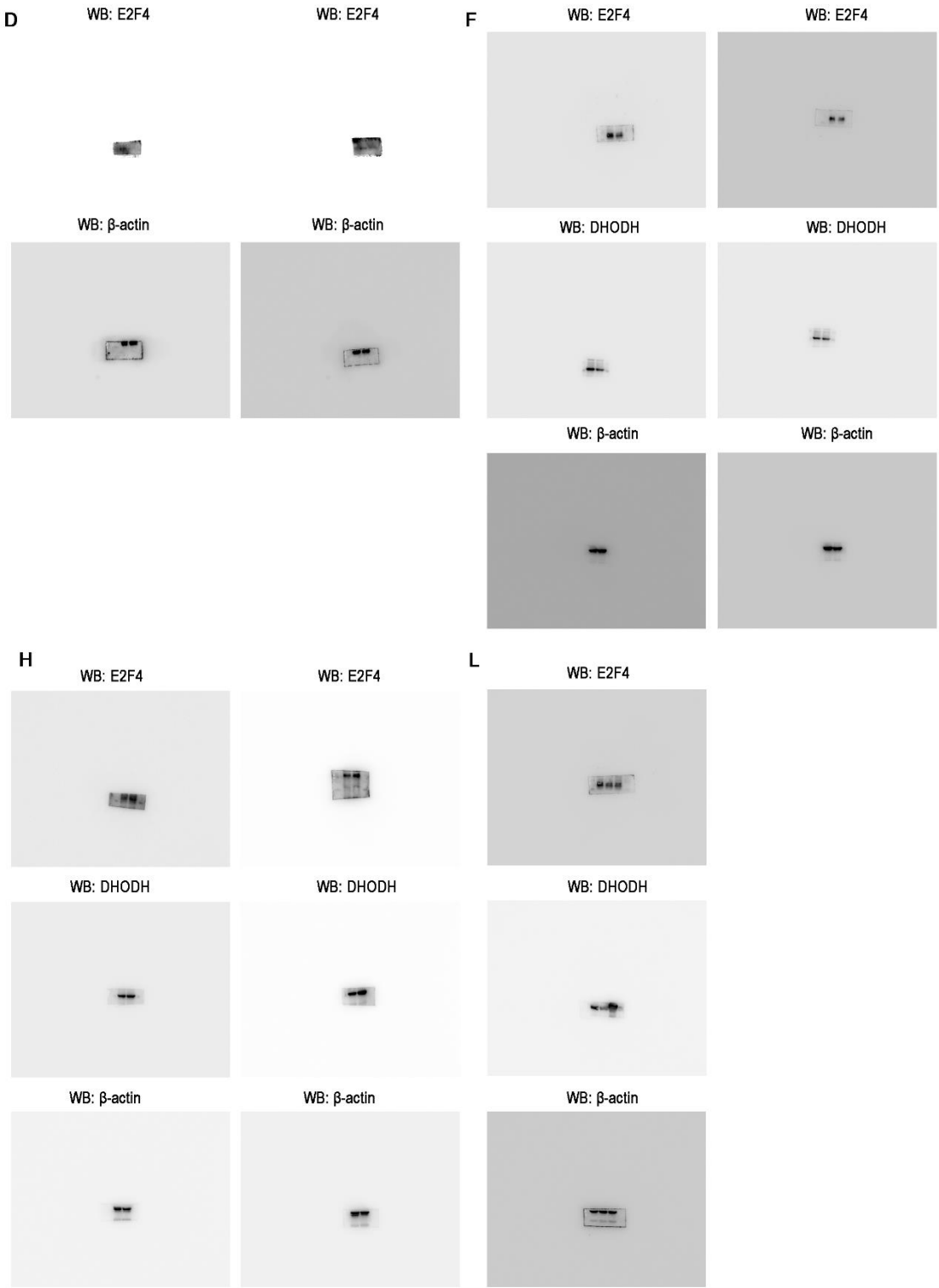

**Figure 6**

**G**

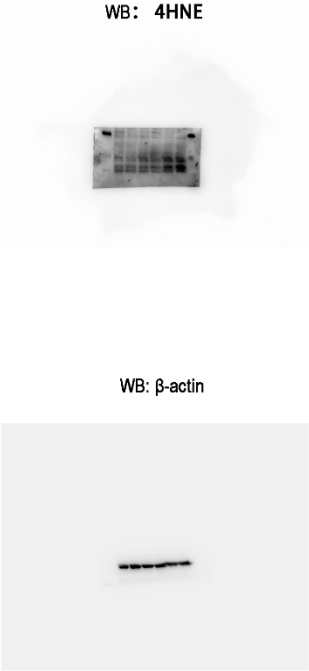

Figure S1

F

WB: POLQ

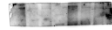

WB: E2F4

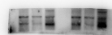

WB:  $\beta$ -actin

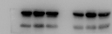

Supplement: Supplementary file 1 — Original Western Blots [file 41419_2024_6618_MOESM1_ESM.pdf]
