# Supplementary material for: POLQ inhibition attenuates the stemness and ferroptosis resistance in gastric cancer cells via downregulation of dihydroorotate dehydrogenase
Source: Cell Death Dis. 2024 Apr 4;15(4):248. doi: 10.1038/s41419-024-06618-5 (PMC10995193; doi:10.1038/s41419-024-06618-5)
Supplement: Supplementary file 3 — Supplementary material [file 41419_2024_6618_MOESM3_ESM.docx]

Supporting Information

**POLQ inhibition attenuates the stemness and ferroptosis resistance in gastric cancer cells via downregulation of dihydroorotate dehydrogenase**

*Yanmei Peng^1†^, Wenbo Zheng^1†^, Yuehong Chen^1^, Xuetao Lei^1^, Zhijing Yang^1^, Yuxuan Yang^1^, Weiqi Liang^1^, Kai Sun^1^*, Guoxin Li ^1^*, Jiang Yu ^1^**

This file includes: Tables S1 and Figure S1

Table S1. Primers used for qRT-PCR

| Gene | Primer | Sequence (5’→3’) |
| --- | --- | --- |
| *CD44* | Forward  Reverse | CTGCCGCTTTGCAGGTGTA CATTGTGGGCAAGGTGCTATT |
| *CD24* | Forward  Reverse | CTCCTACCCACGCAGATTTATTC AGAGTGAGACCACGAAGAGAC |
| *AQP5* | Forward  Reverse | GCCACCTTGTCGGAATCTACT GGCTCATACGTGCCTTTGATG |
| *ALDH1A1* | Forward  Reverse | GCACGCCAGACTTACCTGTC CCTCCTCAGTTGCAGGATTAAAG |
| *MKI67* | Forward  Reverse | ACGCCTGGTTACTATCAAAAGG CAGACCCATTTACTTGTGTTGGA |
| *ATP4B* | Forward  Reverse | CGCCTTCCTAGCAGGCTAC CAGCATATCTGCCGTGAACTT |
| *MUC6* | Forward  Reverse | CTGCCCTATACCAGCAATGGA CTGACCCATGTACTTCCGCTC |
| *GIF* | Forward  Reverse | CAGAGGAAGGTTACAGATCCCT ATGGCTCAGGTGTTACAGAGA |
| *POLQ* | Forward  Reverse | ACTTTTGCTGACCAAGATTTGCT  ACTCATGCCAACGATTTGCAC |
| *PTGS2* | Forward  Reverse | CGGTGAAACTCTGGCTAGACAG  GCAAACCGTAGATGCTCAGGGA |
| *E2F4* | Forward  Reverse | ATCGGGCTAATCGAGAAAAAGTC  TGCTGGTCTAGTTCTTGCTCC |
| *DHODH* | Forward  Reverse | GTTCTGGGCCATAAATTCCGA  TCTGGGTCTAGGGTTTCCTTC |
| *β-actin* | Forward  Reverse | TGGCACCCAGCACAATGAA  CTAAGTCATAGTCCGCCTAGAAGCA |
| *DHODH-CHIP* | Forward  Reverse | AGCTGATGGCGGGAACACTG  GCCGTTGGAGAATAGCCTCTGT |

**Figure S1**


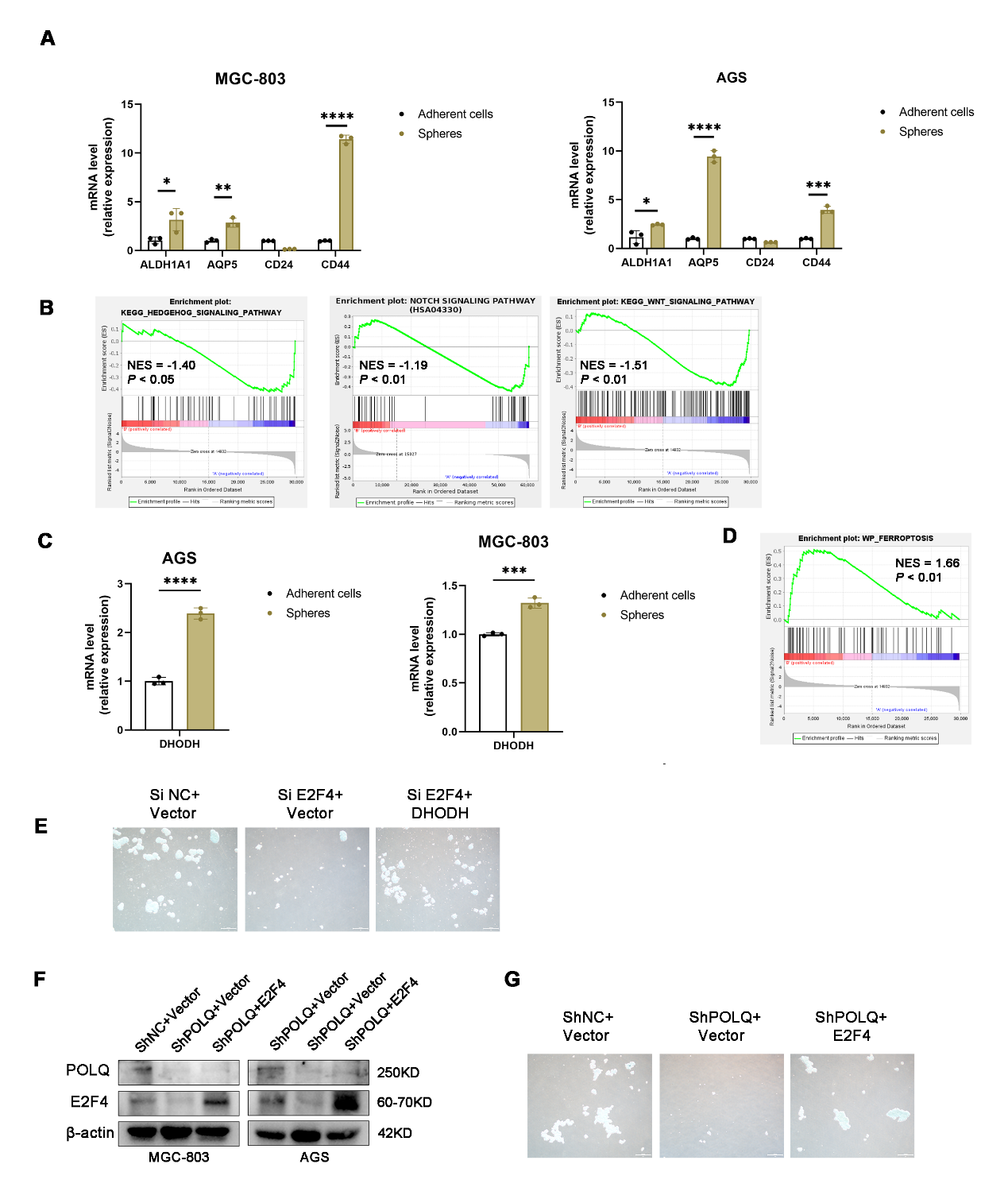


**Figure S1. POLQ mediates stemness of gastric cancer cells by regulated E2F4-DHODH axis.** (A) The levels of gastric cancer stem cell markers were detected in adherent cells and spheres derived from AGS and MGC-803 cells using qRT-PCR. (B) Gene-set enrichment analysis (GSEA) of the RNA-seq profiles between MGC-803-ShPOLQ (B) and MGC-803-ShNC (A) cell lines. (C) The levels of DHODH were detected in adherent cells and spheres derived from AGS and MGC-803 cells using qRT-PCR. (D) Gene-set enrichment analysis (GSEA) of the RNA-seq profiles between MGC-803-ShPOLQ (B) and MGC-803-ShNC (A) cell lines. (E) Sphere formation in control (Si NC), E2F4 knockdown (Si E2F4), and Si E2F4 + DHODH cells was analyzed using oncosphere-initiating medium (scale bar = 200 μm). (F) Protein expression levels in POLQ- knockdown cells overexpressing E2F4. (G) Sphere formation in control (ShNC), POLQ knockdown (ShPOLQ), and ShPOLQ + E2F4 cells was analyzed using oncosphere-initiating medium (scale bar = 200 μm).
